# A Narrative Review of Treatment Options for Patients with Node-Positive Disease After Radical Prostatectomy: Current Evidence and Controversies

**DOI:** 10.3390/cancers17172792

**Published:** 2025-08-27

**Authors:** Paolo Zaurito, Andrea Cosenza, Leonardo Quarta, Pietro Scilipoti, Mattia Longoni, Alfonso Santangelo, Alessandro Viti, Abigail Gettman, Francesco Barletta, Simone Scuderi, Vito Cucchiara, Armando Stabile, Francesco Montorsi, Alberto Briganti, Giorgio Gandaglia

**Affiliations:** 1 Division of Experimental Oncology/Unit of Urology, Urological Research Institute, IRCCS Ospedale San Raffaele, 20132 Milan, Italy; 2 Vita-Salute San Raffaele University, 20132 Milan, Italy; 3 Department of Surgery, University of Michigan, Ann Arbor, MI 48109, USA

**Keywords:** prostate cancer, oncological outcomes, nodal positive disease, radical prostatectomy, treatment options

## Abstract

**Purpose of Review**: In approximately 10–15% of patients with prostate cancer (PCa), pathological lymph node metastases (pN1) are detected at radical prostatectomy (RP). The aim of this review is to describe the various treatment options for pN1 patients, with a focus on the most recent evidence reported in the literature. **Evidence Synthesis**: Due to the lack of prospective studies, several retrospective analyses were conducted according to different types of treatment. Most common strategies are represented by observation plus early salvage radiotherapy (RT) in case of PSA rising, adjuvant androgen deprivation therapy (ADT) alone, or adjuvant RT with or without ADT. Patients with pN1 disease and favorable disease characteristics (lower T stage and ISUP ≤ 2 at RP, <3 metastatic nodes at pathology) have a similar overall mortality risk if observed with PSA testing and eventual use of early salvage RT compared to patients directly treated with adjuvant RT with or without ADT. While conflicting results in terms of survival benefit were reported for the use of adjuvant ADT only, several studies showed an overall survival benefit in patients with pN1 disease treated with adjuvant RT when high-risk features (such as an increasing number of positive nodes, ISUP > 3) were detected at RP. Lastly, few studies analyzed the rate of adverse events following adjuvant ADT or RT, leaving the issue of treatment-related side effects still open. **Summary**: There is no clearly established standard of care for men with pN1 PCa, and disease characteristics should guide the choice of optimal post-operative management for these patients. Prospective data and clinical trials are clearly needed to define the most effective therapeutic strategy.

## Simple Summary

Approximately 15% of prostate cancer patients undergoing radical prostatectomy with lymph node removal are found with nodal metastases, increasing recurrence and mortality risk. This review examines the recent evidence on optimal post-operative treatments, weighing potential benefits against the associated treatments’ toxicities. Adjuvant androgen deprivation (ADT) therapy alone shows limited and inconsistent benefits, while adjuvant radiotherapy, with or without ADT, improves survival in high-risk patients with nodal metastases. In contrast, observation with PSA monitoring seems a suitable approach for low-risk cases, balancing the potential toxicities of adjuvant treatments. In summary, evidence guiding the management of patients with nodal metastases after radical prostatectomy is limited; only one randomized trial was conducted in the pre-PSA era, while the majority of available data are from retrospective studies. Current guidelines emphasize personalizing treatment based on disease characteristics, favoring observation with early salvage radiotherapy in low-risk patients, and reserving adjuvant radiotherapy with or without ADT for those with high-risk features. Risk stratification according to nodal burden, ISUP grade, and tumor stage is therefore critical to balance oncologic control with treatment-related toxicities. Prospective trials are urgently needed to define the optimal post-operative strategy for these patients.

## 1. Introduction

Pathological lymph node metastases (LNs) are identified in approximately 15% of prostate cancer patients treated with radical prostatectomy (RP) and pelvic lymph node dissection [1]. Since the presence of LN metastases at RP is associated with an increased risk of disease recurrence and prostate cancer-specific mortality, the correct management of patients with positive nodes (pN1) is crucial [2].

Different treatment options have been proposed during the last decades, ranging from the use of adjuvant androgen deprivation therapy (ADT) alone to the combination of adjuvant radiotherapy (RT) with or without ADT after RP [3,4]. However, since pN1 patients may present with heterogeneous disease features (i.e., a different number of LN metastases, International Society of Urological Pathology [ISUP] score, or T stage at RP), it is nowadays increasingly needed to select these patients for treatments based on their individual risk, also considering the significant side effects of adjuvant therapies [5,6].

Our aim was to perform a narrative literature review of recent studies evaluating the current evidence on best treatment options for pN1 patients after RP, to explore their oncological outcomes and the evidence on the potentially alternatives to adjuvant treatments.

## 2. Materials and Methods

We performed a narrative literature review search of PubMed for original articles using Medical Subject Headings (MeSH) indexes, keyword searches, and publication types from inception until May 2025. The search was limited to articles in English. The search terms included the following: ‘Prostate cancer’, ‘Radical prostatectomy’, ‘Pathological node positive disease’, and ‘Treatment options’. Relevant articles identified in the reference lists of the selected manuscripts have also been included. Results for pN1 patients from prospective and retrospective studies and randomized controlled trials were reported. The population, intervention, comparison, outcomes, and study design (PICOS) were used to define the inclusion criteria [7] (Table 1). The articles that had been identified were screened separately and then independently selected by two authors (P.Z. and A.C.) who eventually convened on the ones that are included in the present review. Disagreements were solved via a third vote whenever required (G.G.). Abstracts and non-English articles were excluded, as well as review articles, meta-analyses, guidelines, case reports, case series, editorials, and book chapters.

In cases where survival estimates were not reported in the text, we used WebPlotDigitizier v4.6 to digitize Kaplan–Meier curves and extract the 10-year survival estimates for the outcomes of interest [8,9].

## 3. Evidence Synthesis

### 3.1. Oncological Outcomes of pN1 Patients

The oncological outcomes of patients with LN metastases after RP widely range across studies and depend on several clinical and pathological factors. Indeed, the 10-year cancer-specific survival (CSS) in patients with pN1 disease after RP showed considerable variability across retrospective series, ranging from 72% to 98% [10,11]. Similarly, the 10-year overall survival (OS) dropped to approximately 60–70% in patients with high-volume nodal disease (i.e., >2 positive nodes at pathology), suggesting an influence of nodal burden on long-term prognosis [12,13]. Adverse pathological features, such as seminal vesicle invasion (SVI), ISUP groups 4–5 at RP, or the presence of positive surgical margins (PSMs), have also been independently associated with worse oncologic outcomes, regardless of nodal involvement [14]. Within this high-risk setting, the number and volume of nodal metastases are strongly associated with survival in pN1 patients [11]. Among the numerous clinical and pathological characteristics evaluated in pN1 patients, the ISUP group and the number of LN metastases appear to be the most informative prognostic factors for both cancer-specific and overall mortality [11,15]. Due to the heterogeneity of prognosis according to both the number of LN metastases and other adverse pathological features, several treatment options have been implemented for pN1 patients and may include observation, adjuvant ADT alone, and adjuvant RT with or without ADT [16].

### 3.2. Adjuvant ADT Alone

The role of adjuvant ADT alone in men with pN1 disease has been extensively explored over the past decades [17,18,19,20,21,22], yet there is only one randomized clinical trial (RCT) providing evidence in this setting [17]. In this trial, 98 patients with pN1 disease after RP were randomized 1:1 from 1988 to 1993 to receive either adjuvant ADT (n = 47) or deferred ADT (i.e., at the time of recurrence, n = 51). At a median follow-up of nearly 12 years, patients receiving adjuvant ADT had a significant benefit in terms of 10-year progression free-survival (PFS) compared to patients who received deferred ADT (70% vs. 16%; HR: 3.4, 95%CI: 1.96–5.98, *p* = 0.04), and in terms of 10-year OS (76% vs. 53%, HR: 1.84, 95% CI 1.01–3.35, *p* = 0.04). Even more striking was the impact on 10-year CSS (83% in the adjuvant ADT group vs. 51% in the deferred ADT group, *p* = 0.0004). These findings primarily showed that adjuvant ADT could delay disease progression and reduce mortality in pN1 patients. However, several limitations need to be addressed. The small sample size, as well as the lack of stratification by nodal burden, and the absence of modern staging tools such as PSMA-PET imaging limit its generalizability to current clinical practice. Moreover, the study was initiated in the pre-PSA era, and, accordingly, many patients in the observation arm likely received delayed salvage treatments. All these limitations notably affected results.

Retrospective series showed conflicting results regarding the benefit of adjuvant ADT alone in pN1 patients after RP. For instance, several retrospective studies based on large European and North American cohorts reported an inferior benefit in terms of PFS, CSS, and OS for adjuvant ADT alone compared to adjuvant RT with ADT [18,19]. Moreover, Park et al. described no survival benefits for adjuvant ADT alone compared to observation in a cohort of 40 pN1 patients from Korea [21]. The 5-year CSS and OS were 83% and 72% in the adjuvant ADT group vs. 86% and 72% in the observation group, respectively (*p* > 0.05). Similar findings were reported by Wong et al., who showed no significant difference in OS between the use of adjuvant ADT alone and observation in 731 pN1 patients identified through the SEER database [22]. However, a major limitation of all these studies is the absence of key modern intermediate endpoints, such as metastasis-free survival (MFS), and the infrequent reporting of long-term CSS. Indeed, only two studies have provided the 10-year CSS estimates, reducing the strength of long-term conclusions on the effectiveness of adjuvant ADT monotherapy for pN1 patients [17,23]. A summary of the baseline characteristics and oncological outcomes of the included studies is presented in Table 2 and Table 3.

In summary, the current body of evidence on adjuvant ADT alone is largely based on older studies with inconsistent results between the only RCT published and retrospective series in support of adjuvant ADT alone, as well as limited long-term oncologic endpoints. More recent prospective data are needed to clarify its role, especially in comparison to combination strategies that include RT. The strength of the EAU 2025 recommendation—which suggests to considered adjuvant ADT monotherapy in pN1 patients with multiple high-risk features—remains weak, reflecting the limited high-level evidence available [24].

**Table 2:** Summary of the baseline characteristics of patients with prostate cancer and LN metastases after RP according to the type of treatment received.

Authors and Year	Patients with LNI (n)	Treatment Period (Years)	ISUP Score at Pathology	pT Stage	PSM	Treatment Groups	Nodes Removed (Nodes Positive)	Primary Outcome
Messing et al. (2006) [17], Multicenter RCT	98	1988–1993	ISUP 1: 34, 35%; ISUP 2–3: 46, 47%; ISUP 4–5: 18, 18%	pT3b:59, 60%	63 (64%)	Adjuvant ADT vs. Observation	11 (2) for adjuvant ADT group vs. 14 (2) for observation group	CSS; OS
Park et al. (2011) [21], Single center retrospective	40	1990–2008	ISUP 1: 2, 5%; ISUP 2–3: 7, 18%; ISUP 4–5: 31, 77%	pT2: 2, 5%; pT3a: 11, 28%; pT3b: 27, 67%	27 (67%)	Adjuvant ADT vs. Observation	-	PFS; CSS; OS
Wong et al. (2009) [22], Multicenter retrospective	731	1991–1999	ISUP 4–5: 379, 52%	pT2: 209 (29%); pT3a: 167 (23%); pT3b–pT4: 355, 48%)	-	Adjuvant ADT vs. Observation	-	OS
Abdollah et al. (2014) [23], Multicenter retrospective	1107	1988–2010	ISUP 1: 155, 14%; ISUP 2–3: 518, 47%; ISUP 4–5: 434, 39%	pT2–pT3a: 351, 32%; pT3b: 681, 62%; pT4: 75, 6%	657 (59%)	Adjuvant ADT vs. Adjuvant RT with ADT	14 (1) overall	OS
Bianchi et al. (2018) [25], Multicenter retrospective	518	1995–2015	ISUP 1: 34, 7%; ISUP 2–3: 203, 39%; ISUP 4–5: 281, 54%	pT2–pT3a: 176, 34%; pT3b: 304, 59%; pT4: 38, 7%	309 (60%)	Adjuvant ADT vs. Adjuvant RT with ADT	15 (2)	OS
Bravi et al. (2020) [26], Single center retrospective	372	1991–2017	ISUP 1–3: 161, 43%; ISUP 4–5: 211, 57%	pT2–pT3a: 122, 33%; pT3b–pT4: 250, 67%	203 (55%)	Adjuvant RT vs. Adjuvant RT with ADT	-	CSS; OS
Tilki et al. (2017) [18], Single center retrospective	773	2005–2013	ISUP 1–3: 478, 62%; ISUP 4–5: 293, 38%	pT2: 72, 9%; pT3a: 161, 21%; pT3b: 539, 70%	423 (55%)	Adjuvant RT with or without ADT vs. Adjuvant ADT vs. Observation	15 (1)	BCR; PFS
Touijer et al. (2018) [27], Multicenter retrospective	1388	1988–2010	ISUP 1: 157, 11%; ISUP 2–3: 670, 49%; ISUP 4–5: 552, 40%	pT3a: 536, 39%; pT3b: 752, 54%; pT4: 100, 7%	734 (53%)	Adjuvant RT with ADT vs. Adjuvant ADT vs. Observation	-	CSS; OS
Touijer et al. (2014) [13], Single center retrospective	369	1988–2010	ISUP 1: 6, 2%; ISUP 2–3: 178, 48%; ISUP 4–5: 185, 50%	pT2: 46, 12%; >pT2: 323, 88%	138 (37%)	Observation/ Salvage RT	15 (-)	CSS; OS
Tilki et al. (2022) [28], Single center retrospective	1614	1995–2017	-	pT2–pT3a: 15,647 (87%); pT3b-pT4: 2266 (13%) *	3277 (18%) *	Adjuvant RT with or without ADT vs. Observation/ Salvage RT	12 (1) *	OS
Marra et al. (2025) [29], Multicenter retrospective	1103	2000–2021	ISUP 1–3: 433, 39%; ISUP 4–5: 670, 61%	pT2-pT3a: 428 (39%); pT3b-pT4: 675 (61%)	554 (50%)	Adjuvant RT with or without ADT vs. Observation/ Salvage RT	19 (1)	OS

* Results reported for overall population (pN0 and pN1). **Abbreviations**: Radical prostatectomy (RP); lymph node invasion (LNI); lymph node metastases (LNs); radiotherapy (RT); androgen deprivation therapy (ADT), International Society of Urological Pathology (ISUP).

**Table 3:** Summary of the oncological outcomes of patients with prostate cancer and LN metastases after RP according to the type of treatment received.

Study	Enrolled Patients n, (%)	Follow-Up	Events n, (%)	BCR-FS	PFS	CSS	OS
Messing et al. (2006) [17], Multicenter RCT *	Overall 98, Adjuvant ADT 47 (48), Observation 51 (52)	11.9 years (9.7–14.5)	Progression: 66 (67) Death from PCa: 32 (33) Death from other cause: 13 (13)	-	10 yr Adjuvant ADT (70%) vs. Observation (16%), *p* < 0.001	10 yr Adjuvant ADT (85%) vs. Observation (51%), *p* = 0.0004	10 yr Adjuvant ADT (76%) vs. Observation (53%), *p* = 0.04
Park et al. (2011) [21], Single center retrospective	Overall 40, Adjuvant ADT 18 (45), Observation 22 (55)	55.7 months	Progression: 15 (37) Death from PCa: 6 (15) Death from other cause: 4 (10)	-	5 yr Adjuvant ADT (72%) vs. Observation (77%), *p* = 0.65	5 yr Adjuvant ADT (83%) vs. Observation (86%), *p* < 0.05	5 yr Adjuvant ADT (72%) vs. Observation (72%), *p* < 0.05
Wong et al. (2009) [22], Multicenter Retrospective *	Overall 731, Adjuvant ADT 209 (29), Observation 522 (71)	-	Death from PCa: 71 (10) Death from any cause: 269 (37)	-	-	10 yr Adjuvant ADT (90%) vs. Observation (90%), *p* > 0.05	10 yr Adjuvant ADT (58%) vs. Observation (58%), *p* > 0.05
Abdollah et al. (2014) [23], Multicenter retrospective	Overall 1107, Adjuvant ADT 721 (65), Adjuvant ADT + RT 386 (35)	7.1 years	-	-	-	10 yr Adjuvant ADT (82%) vs. Adjuvant RT with ADT (87%), *p* < 0.05	8 yr Adjuvant ADT (75%) vs. Adjuvant RT with ADT (88%), *p* < 0.001
Bianchi et al. (2018) [25], Multicenter retrospective	Overall 518, Adjuvant ADT 218 (42), Adjuvant ADT + RT 300 (58)	52 (30–84) months	-	-	-	8 yr Adjuvant ADT (69%) vs. Adjuvant RT with ADT (72%), *p* = 0.6	-
Bravi et al. (2020) [26], Single center retrospective	Overall 372, Adjuvant RT without ADT 100 (27) vs. Adjuvant RT with ADT 272 (73)	77 (44–113) months	Progression: 77 (21) Death from PCa: 18 (5) Death from other any cause: 48 (13)	-	10 yr Adjuvant RT without ADT (92%) vs. Adjuvant RT with ADT (70%), *p* = 0.029	10 yr Adjuvant RT without ADT (98%) vs. Adjuvant RT with ADT (92%), *p* = 0.11	10 yr Adjuvant RT without ADT (81%) vs. Adjuvant RT with ADT (85%), *p* = 0.8
Tilki et al. (2017) [18], Single center retrospective	Overall 773, Adjuvant RT with or without ADT 268 (34) vs. Observation /Salvage RT 505 (66)	33.8 (17.1–49) months	-	4 yr Adjuvant RT with or without ADT (57%) vs. Observation /Salvage RT (43%), *p* < 0.001	4 yr Adjuvant RT with or without ADT (92%) vs. Observation /Salvage RT (83%), *p* < 0.05	**-**	-
Touijer et al. (2018) [27], Multicenter Retrospective *	Overall 1388, Adjuvant RT with ADT 325 (23) vs. Adjuvant ADT 676 (49) vs. Observation /Salvage RT 387 (28)	69 (36–126) months	Death from PCa: 171 (12) Death from other any cause: 321 (23)	**-**	-	10 yr Adjuvant RT with ADT (86%) vs. Adjuvant ADT (82%) vs. Observation /Salvage RT (68%), *p* < 0.001	10 yr Adjuvant RT with ADT (75%) vs. Adjuvant ADT (69%) vs. Observation /Salvage RT (65%), *p* < 0.001
Touijer et al. (2014) [13], Single center retrospective	Overall 369 (100)	4 years	Progression: 70 (19) Death from PCa: 37 (10) Death from other any cause: 64 (17)	10 yr Observation /Salvage RT (28%)	10 yr Observation /Salvage RT (65%)	10 yr Observation /Salvage RT (72%)	10 yr Observation /Salvage RT (60%)
Tilki et al. (2022) [28], Single center retrospective	Overall 1614, Adjuvant RT with or without ADT 412 (26) vs. Observation /Salvage RT 1202 (74)	7 years	-	-	-	-	7 yr Adjuvant RT with or without ADT (92%) vs. Observation /Salvage RT (77%), *p* = 0.03 **
Marra et al. (2025) [29], Multicenter retrospective	Overall 1103, Adjuvant RT with or without ADT 488 (44) vs. Observation /Salvage RT 615 (56)	51 (26–87) months	Death from PCa: 39 (4) Death from other any cause: 44 (4)	-	-	**-**	7 yr Adjuvant RT with or without ADT (92%) vs. Observation /Salvage RT (84%), *p* = 0.006 ***

* Estimates provided using WebPlotDigitizier v4.6 to digitize Kaplan–Meier curves for the outcomes of interest. ** Estimates provided only for men with four or more positive LN metastases (high-risk mortality group). *** Estimates provided only for men in the high-risk mortality group (pT3b–T4 and/or ISUP 4–5 at RP, ≥2 LN metastases). **Abbreviations**: Radical prostatectomy (RP); biochemical recurrence-free survival (BCR-FS); progression-free survival (PFS); cancer-specific survival (CSS); overall survival (OS); lymph node metastases (LNs); androgen deprivation therapy (ADT); radiotherapy (RT); randomized control trial (RCT).

### 3.3. Adjuvant RT with or Without ADT

The role of adjuvant RT with or without adjuvant ADT in men with pN1 disease has been explored across several retrospective series, but lacks evidence from RCTs [18,19,25,26,27,30,31,32]. Across retrospective cohorts, most patients selected for adjuvant RT exhibited adverse pathological features, such as ISUP ≥ 3, more than two LN metastases, pT3–4 disease, and PSM.

Abdollah et al. analyzed 1107 pN1 patients treated with RP between 1988 and 2010. All patients received adjuvant ADT, whereas adjuvant RT was received only by 35% (n = 386) of patients [19]. The median follow-up was 7 years. Patients were stratified according to a regression-tree analysis in five different risk groups for cancer-specific mortality (CSM), accounting for the number of LN metastases, pathologic ISUP and T stages, and surgical margin status. The use of adjuvant RT plus ADT was associated with reduced CSM rates compared to adjuvant ADT alone in patients with ≤2 LN metastases, ISUP 2–5, pT3b/pT4 stage, or PSM (i.e., intermediate-risk group, HR: 0.30, *p* = 0.002), and in patients with 3–4 LN metastases, regardless of disease characteristics (i.e., high-risk group, HR: 0.21, *p* = 0.02). Conversely, all other patients with LN metastases in this cohort seem they did not benefit significantly from adjuvant RT with ADT. These findings stressed how the survival benefit related to adjuvant RT is strongly related to the number of LN metastases and tumor characteristics. Conversely, Bianchi et al. found no difference in terms of 8-year CSM among pN1 patients from a multi-institutional European cohort treated with RP between 1995 and 2015 and then received adjuvant ADT with or without RT (72% vs. 69%, *p* = 0.6) [25]. Moreover, they failed to validate the benefit of the risk stratification tool for pN1 patients previously proposed by Abdollah et al. [19], leaving questions open about the optimal treatment strategy for this patient population.

In this context, Bravi et al. evaluated 372 pN1 patients treated between 1996 and 2016 at a single institution and compared the use of adjuvant RT with or without adjuvant ADT in term of CSS and OS [26]. There were 272 (73%) patients that received adjuvant RT with ADT, whereas 100 patients (27%) received only adjuvant RT. In this series, the addition of adjuvant ADT did not significantly improve CSS (HR: 0.91, *p* = 0.8) and OS (HR: 5.39, *p* = 0.11), suggesting that adjuvant RT alone may be sufficient for pN1 patients. Conversely, different results were reported in other retrospective cohorts and were confirmed by a recent metanalysis, which reported a CSS and OS benefit in pN1 patients treated with adjuvant RT plus ADT compared to adjuvant ADT alone [30,31,32]. However, the heterogeneity of pN1 patients, as well as the LN metastasis disease burden, were strong predictors of the treatment success for all retrospective series, indicating a possible selection bias.

Comparative studies further stress the use of adjuvant RT. Tilki et al. analyzed 773 pN1 patients treated between 2005 and 2013, comparing 268 (34%) patients who received adjuvant RT with or without ADT, with 505 (66%) patients initially observed [18]. The authors reported a 4-year BCR-free survival of 43% for the observation group vs. 57% for the adjuvant RT with ADT group, respectively (*p* < 0.001). The 4-year MFS rate was 83% for the observation group vs. 92% for the adjuvant RT with ADT group, respectively (*p* < 0.05). However, this study did not investigate any differences in terms of CSS and OS. Lastly, Touijer et al. analyzed 1338 pN1 patients treated with RP between 1988 and 2010, which were either observed (n = 387, 29%), received adjuvant ADT alone (n = 676, 50%), or had adjuvant RT with ADT (n = 325, 23%) [27]. After a median follow-up of 69 months, the use of adjuvant RT plus ADT was associated with a higher 10-year OS compared to ADT alone (HR: 0.46, 95% CI: 0.32–0.66, *p* < 0.05) or observation (HR: 0.41, 95% CI: 0.27–0.64, *p* < 0.05). Similar results were reported for 10-year CSS (all *p* < 0.001). After stratifying pN1 patients in three risk groups according to ISUP and T stage at RP, the number of positive nodes (1 vs. 2 vs. >2), and surgical margin status, authors found a significant higher risk of 10-year overall mortality (OM) for higher-risk pN1 patients compared to the low-risk group, with the former group having the highest benefit from the use of adjuvant RT plus ADT.

### 3.4. Observation with PSA Monitoring

Recent evidence supports observation for pN1 patients with low-risk pathological features using PSA monitoring [11,16]. Only one RCT involved observation as a treatment option for pN1 patients treated between 1988 and 1993 [17], showing a significant benefit for adjuvant ADT alone vs. observation. As previously discussed, several critical issues limited the clinical application of these results from the pre-PSA era.

Due to the lack of other RCTs, only retrospective studies analyzed the oncological outcomes of pN1 patients observed with PSA monitoring after RP [12,13,33,34]. Toujer et al. analyzed 369 pN1 patients from a single institution that were followed with observation until BCR (defined as PSA > 0.1 ng/mL with confirmatory rise), and reported a 10-year OS of 60%, and a 10-year CSS of 72% for the overall population, respectively [13]. Patients with adverse pathology features (ISUP 4–5 and more than three LN metastases) had a higher risk of BCR compared to patients with a low number of LN metastases, suggesting the possibility of identifying a favorable prognostic group among pN1 patients. Similarly, other retrospective studies showed that the total number of LN metastases identified at pathology, as well as the presence of ISUP 4–5 at RP, and the diameter of the largest LN metastases were all independent predictors of BCR and clinical recurrence in pN1 patients that were observed [12,33,34]. The 5-year BCR-free survival ranged 27–28% across studies [33]. Critical issues of these studies included small sample sizes, the lack of mention of salvage treatment criteria [33], or offering salvage treatment only in cases of clinical [34] or symptomatic progression [12]. All these issues may have affected the survival estimates reported.

Focusing on observation, Tilki et al. analyzed a single-center retrospective cohort of 17,913 patients treated with RP between 1997 and 2017 and compared the use of adjuvant RT with or without ADT vs. observation plus salvage RT after surgery. A total of 1614 (9%) patients had pN1 disease (1323 [82%] patients with 1–3 LN metastases, 291 [18%] with ≥4 LN metastases) [28]. At a median follow-up of nearly 7 years, 412 (26%) patients received adjuvant RT (mostly without adjuvant ADT) and 1202 (74%) patients underwent observation plus early salvage RT (defined as RT started with PSA values ≤ 0.5 ng/mL). The use of adjuvant RT was associated with a lower OM risk per unit increase in metastatic LNs (HR: 0.92, 95% CI, 0.85 to 0.99, *p* = 0.03). Moreover, patients with more than four LN metastases treated with early salvage RT have a higher risk of 7-year OM compared to those treated with adjuvant RT after surgery (23% vs. 7%, respectively, *p* < 0.05), whereas patients with 1–3 LN metastases treated with salvage RT have similar 7-yr OM estimates compared to those treated with adjuvant RT (14% vs. 14%, *p* > 0.05), suggesting the possibility of stratifying pN1 patients to observation and early salvage RT considering the number of LN metastases. The single-center data, as well as the inclusion of patients back to 1997 and of those with PSA persistence after RP, represent possible limitations of this study. To address these concerns, Marra et al. reported the results of a multicenter contemporary cohort of pN1 patients treated with RP that received either adjuvant RT or observation plus early salvage RT from 2000 to 2021 [29]. A total of 1103 pN1 patients were identified, of whom 488 (44%) received adjuvant RT and 615 (56%) observation plus eventual early salvage RT. The authors created an individual risk score for OS based on significant predictors for mortality such as the T stage, ISUP at RP, and number of LN metastases. Patients were then stratified into a low/intermediate-risk (1–2 points) group vs. high-risk group (>2 points). At a median follow-up of 51 months, patients receiving adjuvant RT had a higher OS benefit in the high-risk group (92%; 95% CI, 87–96%) compared to the use of observation and early salvage RT (84%, 95% CI, 77–90%, *p* = 0.006). No differences were detected in OS for men treated with adjuvant RT vs. observation for the overall population and for the low/intermediate-risk group. The positive impact of adjuvant RT in men with high-risk features was also confirmed after excluding men with PSA persistence after surgery. These findings further support the use of adjuvant RT for pN1 patients with high-risk pathology features at RP (i.e., pT3/T4 stage, ISUP 4–5, or ≥3 LN metastases).

### 3.5. Adverse Events of Adjuvant Treatments

The need to personalize the treatment of pN1 patients according to disease characteristics, and adopt an observation approach if feasible, is further underscored by the side effects of adjuvant treatments [6,35,36]. The use of whole-pelvis radiotherapy (WPRT) in pN1 patients is characterized by a high incidence of early and late toxicities compared to prostate-only RT. A recent RCT reported, at a follow-up of 68 months, a higher incidence of grade II genito-urinary (GU) toxicities for patients treated with WPRT compared to prostate RT (20% vs. 8%, *p* = 0.02) [35]. Similar to RT, the use of adjuvant ADT is also characterized by a higher incidence of side effects. Indeed, cardiovascular events are a well-known significant cause of death for patients with locally advanced PCa, and the use of novel Androgen Receptor Pathway Inhibitors (ARPIs) showed, in clinical trials, an association with a small increase of cardiovascular events [6,37,38,39]. Fatigue, hot flashes, and decreased libido are other side effects usually associated with ADT treatment [40]. However, in the only RCT published in the pN1 setting, adjuvant ADT was well-tolerated, and no patients discontinued the treatment due to toxic effects [17].

## 4. Discussion

Considering all the findings reported, guidelines suggest personalizing the treatment choice of pN1 patients according to disease characteristics, ideally trying to avoid the use of adjuvant treatments, characterized by a relevant number of side effects, in selected patients with low-risk features. Indeed, patients with a low disease burden (<3 positive LNs, ISUP group ≤ 2, and no PSA persistence after surgery) could be managed with observation plus early salvage RT, as suggested by the guidelines [3,4]. The use of adjuvant treatment should be limited to those patients with adverse pathological features, where several studies showed a benefit in terms of OS. There is an increasing need to weigh the potential short- and long-term toxicities of RT and ADT against the possible but modest reduction in the mortality risk when considering their use in men with a low number of LN metastases. These considerations were furthermore supported by a metanalysis of several studies, which concluded that risk stratification according to pathological features is the key to select the optimal post-operative therapy of pN1 patients [11]. To conclude, given the paucity of prospective results on treatment options for pN1 patients, the design of RCTs is still crucial, aiming to better define the best treatment strategy for these patients.

## 5. Limitations

Our work is not devoid of limitations. As this is a narrative review of the literature available on PubMed, no other research databases were consulted. As a consequence, we are not able to ensure comprehensive coverage. As previously noted, only one RCT (published in 2006) compared two treatment arms for pN1 patients, and all the evidence published in recent years comes from retrospective studies, reflecting the heterogeneity that still characterizes the post-operative management of these patients. This heterogeneity is related to selection bias, differences in follow-up duration, confounding factors associated with treatment allocation after RP (e.g., comorbidities and functional status), surgical techniques, and radiotherapy protocols. Furthermore, only two studies have reported 10-year CSS estimates, while another study provided findings only on intermediate endpoints (BCR-FS and MFS), thereby limiting the strength of long-term conclusions regarding the effectiveness of eventual adjuvant treatments in pN1 patients [17,18,19]. As a consequence, causal associations often reported in this context should be interpreted with caution. The use of PSMA PET is currently changing the restaging of pN1 patients, increasing the accuracy for detecting distant disease compared to conventional imaging [41]. However, the optimal PSA level at which to recommend PSMA PET after RP is still under investigation. Finally, the RADICALS-RT results showed no difference in post-RP progression rates between high-risk patients receiving adjuvant RT versus salvage RT, with a lower rate of RT-related side effects in the salvage group [42]. Although these findings support the safe use of salvage RT, it should be noted that only 5% of the trial participants were pN1, which may limit the interpretation of the results for this subgroup.

## 6. Conclusions

Recent evidence suggests the increasing need to stratify patients with pN1 disease after RP according to both the number of LN metastases and primary tumor characteristics. This patient selection is crucial to ensure good oncological control, balancing the side effects related to adjuvant ADT or RT. There is no clearly established standard of care for men with pN1 PCa, and disease characteristics should guide the choice of optimal post-operative management for these patients. Prospective data and clinical trials are needed to define the most effective therapeutic strategy.

## Figures and Tables

**Table 1:** Population, intervention, comparator, outcome, and study design (PICOS) framework and search strategy.

**Clinical question:**	To report the evidence regarding the efficacy and safety of current available treatment options for patients with prostate cancer and LN metastases after radical prostatectomy.
**Population:**	Patients with pN1 disease after radical prostatectomy.
**Intervention:**	Any adjuvant treatment after surgery (adjuvant RT with or without adjuvant ADT, adjuvant ADT alone)
**Comparison:**	Observation plus early salvage RT
**Outcomes:**	BCR-FS, PFS, CSS, OS, CTCAE v5.0
**Study design:**	Prospective and retrospective studies and randomized controlled trials.
**Databases searched:**	PubMed
**Search terms used (including MeSH and keyword text):**	(prostate cancer) OR (PCa) AND (radical prostatectomy) OR (prostatectomy for cancer) AND (pathological node positive disease) OR (LNI) OR (lymph node metastases) AND (treatment options) OR (adjuvant treatments)
**Manual search:**	Relevant citations in identified articles and additional manual examination of articles published in peer-reviewed journals.
**Eligibility criteria:**	English full-text articles published from inception to May 2025, including patients with prostate cancer and LN metastases at radical prostatectomy that were either treated with any form of adjuvant treatment after surgery or observed with PSA testing. Relevant articles identified in the reference lists of the selected manuscripts have also been included.
**Exclusion criteria:**	Review articles, meta-analyses, guidelines, case reports, case series, editorials, and book chapters. Preclinical studies not involving humans. Studies with oncological outcomes not extractable.

**Abbreviations**: Lymph node (LN); radiotherapy (RT); androgen deprivation therapy (ADT); biochemical recurrence-free survival (BCR-FS); progression-free survival (PFS); cancer-specific survival (CSS); overall survival (OS); prostate specific-antigen (PSA); lymph node invasion (LNI); Common Terminology Criteria for Adverse Events (CTCAE).
